# Oral polio revaccination is associated with changes in gut and upper respiratory microbiomes of infants

**DOI:** 10.3389/fmicb.2022.1016220

**Published:** 2022-10-28

**Authors:** Márcia Melo Medeiros, Anna Cäcilia Ingham, Line Møller Nanque, Claudino Correia, Marc Stegger, Paal Skyt Andersen, Ane Baerent Fisker, Christine Stabell Benn, Miguel Lanaspa, Henrique Silveira, Patrícia Abrantes

**Affiliations:** ^1^Global Health and Tropical Medicine (GHTM), Instituto de Higiene e Medicina Tropical (IHMT), Universidade NOVA de Lisboa (UNL), Lisboa, Portugal; ^2^Department of Bacteria, Parasites and Fungi, Statens Serum Institut, Copenhagen, Denmark; ^3^Bandim Health Project, Bissau, Guinea-Bissau; ^4^Bandim Health Project, Odense Patient Data Explorative Network, Institute of Clinical Research, Odense University Hospital/University of Southern Denmark, Odense, Denmark; ^5^Danish Institute for Advanced Study, University of Southern Denmark, Odense, Denmark

**Keywords:** OPV-revaccination, non-specific effects, 16S rRNA deep sequencing, bacterial microbiota, healthier microbiome composition, upper respiratory microbiome, gut microbiome

## Abstract

After the eradication of polio infection, the plan is to phase-out the live-attenuated oral polio vaccine (OPV). Considering the protective non-specific effects (NSE) of OPV on unrelated pathogens, the withdrawal may impact child health negatively. Within a cluster-randomized trial, we carried out 16S rRNA deep sequencing analysis of fecal and nasopharyngeal microbial content of Bissau–Guinean infants aged 4–8 months, before and after 2 months of OPV revaccination (revaccinated infants = 47) vs. no OPV revaccination (control infants = 47). The aim was to address changes in the gut and upper respiratory bacterial microbiotas due to revaccination. Alpha-diversity for both microbiotas increased similarly over time in OPV-revaccinated infants and controls, whereas greater changes over time in the bacterial composition of gut (*p_*adjusted*_* < 0.001) and upper respiratory microbiotas (*p_*adjusted*_* = 0.018) were observed in the former. Taxonomic analysis of gut bacterial microbiota revealed a decrease over time in the median proportion of *Bifidobacterium longum* for all infants (25–14.3%, *p* = 0.0006 in OPV-revaccinated infants and 25.3–11.6%, *p* = 0.01 in controls), compatible with the reported weaning. Also, it showed a restricted increase in the median proportion of *Prevotella*_9 genus in controls (1.4–7.1%, *p* = 0.02), whereas in OPV revaccinated infants an increase over time in Prevotellaceae family (7.2–17.4%, *p* = 0.005) together with a reduction in median proportion of potentially pathogenic/opportunistic genera such as *Escherichia*/*Shigella* (5.8–3.4%, *p* = 0.01) were observed. Taxonomic analysis of upper respiratory bacterial microbiota revealed an increase over time in median proportions of potentially pathogenic/opportunistic genera in controls, such as *Streptococcus* (2.9–11.8%, *p* = 0.001 and *Hemophilus* (11.3–20.5%, *p* = 0.03), not observed in OPV revaccinated infants. In conclusion, OPV revaccination was associated with a healthier microbiome composition 2 months after revaccination, based on a more abundant and diversified bacterial community of Prevotellaceae and fewer pathogenic/opportunistic organisms. Further information on species-level differentiation and functional analysis of microbiome content are warranted to elucidate the impact of OPV-associated changes in bacterial microbiota on child health.

## Introduction

The enormous contribution of vaccines against childhood diseases is well-known (Uwizihiwe, 2015), and much of the decline in child mortality since 1990 (United Nations Inter-agency Group for Child Mortality Estimation [UN IGME], 2019) may be ascribed to the increase in vaccination coverage (National Research Council (US) Working Group on the Effects of Child Survival and General Health Programs on Mortality, 1993). Furthermore, analyses of epidemiological data on childhood mortality have pointed to additional beneficial effects provided by live-attenuated vaccines, such as measles (MV) and Bacillus Calmette-Guérin (BCG) vaccines (Higgins et al., 2016), and, oral polio vaccine (OPV) (Andersen et al., 2018, 2021), by decreasing the all-cause child mortality rate in low income settings (Shann, 2010; Aaby and Benn, 2021; Prentice et al., 2021). This has been confirmed in several randomized clinical trials (Aaby et al., 2010; Lund et al., 2015; Biering-Sørensen et al., 2017). These vaccines seem to confer protection against unrelated pathogens, exhibiting non-specific effects (NSE). The development of a heterologous adaptive host immune response and the so-called, trained immunity or innate immune memory, i.e., epigenetic and metabolic reprogramming of innate immune cells (Netea et al., 2016), have been pointed out as possible immune mechanisms behind NSE. BCG’s capacity to induce a heterologous adaptive immune response and trained immunity has been confirmed in clinical trials with healthy adult volunteers (Kleinnijenhuis et al., 2012, 2014), and a recent retrospective analysis done at Mayo Clinic raised a possible role for trained immunity toward the partial protection provided by OPV against SARS-CoV-2 infection (Pawlowski et al., 2021) and some authors even found that OPV immunization vs. placebo reduced the number of laboratory-confirmed COVID-19 infections (Yagovkina et al., 2022) and improved overall health in males during the COVID-19 pandemic (Fisker et al., 2022). Eradication of polio infection preconizes the withdrawal of OPV from routine immunization programs (World Health Organization [WHO], 2021b). However, considering the beneficial NSE, an increased understanding of them is urgently needed to mitigate the potential impact of OPV withdrawal on child survival in these settings.

Gut commensal bacteria are essential for the development and function of innate and adaptive immune systems (Belkaid and Hand, 2014), and potentially play a role in the induction of innate immune memory (Danelishvili et al., 2019). Polioviruses (PV), including the live-attenuated ones present in OPV formulation, replicate in the oropharyngeal, but mainly in gut mucosal cells (Mendelsohn et al., 1989). The specific composition of gut bacterial microbiota seems to be important to drive a strong OPV-induced immune response. For example, Actinobacteria, mainly represented by *Bifidobacterium longum*, seem to be important to drive specific T-cell and antibody responses to live-attenuated PV (Huda et al., 2014), whilst a more diverse environment with abundance in Firmicutes and Proteobacteria seems to have a negative effect (Zhao et al., 2020). Furthermore, some authors have suggested a role for pathogenic bacteria or environmental enteropathy in the low-immunogenicity levels of oral vaccines in low-income countries (Grassly et al., 2016; Parker et al., 2018, 2021).

The OPV-induced immune response seems to play a role against pathogenic gut bacteria by reducing etiology-specific bacterial diarrhea in male infants (Upfill-brown et al., 2020), and possibly favoring innate immune mechanisms that may mediate OPV-NSE, such as the presence of anti-microbial peptides in the stool of newborns 6 weeks after the first OPV dose administered at birth, concomitantly with BCG (Alam et al., 2015). Previous evidence furthermore suggests that OPV may protect against upper respiratory infections, such as acute otitis media, caused by other viruses from the family Picornaviridae, such as Rhinoviruses, through immune mechanisms involving heterologous adaptive response and antiviral innate defense based on type I interferon (Seppälä et al., 2011). All studies above highlight the complex interaction among OPV, bacteria, and/or viruses present in the respiratory tract and gut. We hypothesized that OPV may induce changes in the microbiome of the sites of live-attenuated PV replication, which may mediate OPV-NSE.

To assess the effect of OPV revaccination on the diversity and composition of the bacterial microbiota of the gut and upper respiratory tract, we carried out the Micro-OPV study. Micro-OPV is a cohort study within a cluster-randomized trial, the RECAMP-OPV (Varma et al., 2019). RECAMP-OPV had a random allocation of infants to OPV revaccination or no revaccination, and it was conducted by the Bandim Health Project (BHP) in Guinea-Bissau. Infants aged 17–34 weeks participating in RECAMP-OPV and living 2 h-drive from the capital Bissau were recruited to the Micro-OPV. At this age range infants have already been eligible for a set of vaccines as part of the routine immunization plan: BCG and first OPV dose at birth (OPV0), followed by three doses of Pentavalent, Pneumococcal-conjugated, and OPV, both administrated at 6, 10, and 14 weeks of age, and Rotavirus vaccine administrated at 6 and 10 weeks of age (World Health Organisation [WHO], 2021a). Due to randomization design, recruited infants shared similar major microbiome determinants which are detailed below (in Materials and methods). Fecal and nasopharyngeal microbial content of infants were collected at enrollment and after 2 months of follow-up and submitted to 16S rRNA deep sequencing analysis. We compared the changes in the gut and upper respiratory bacterial microbiotas from enrollment to follow-up between 47 OPV revaccinated infants and 47 not OPV revaccinated controls, who completed the 2 months of follow-up.

## Materials and methods

### Participants, study design, and settings

**FIGURE 1:**
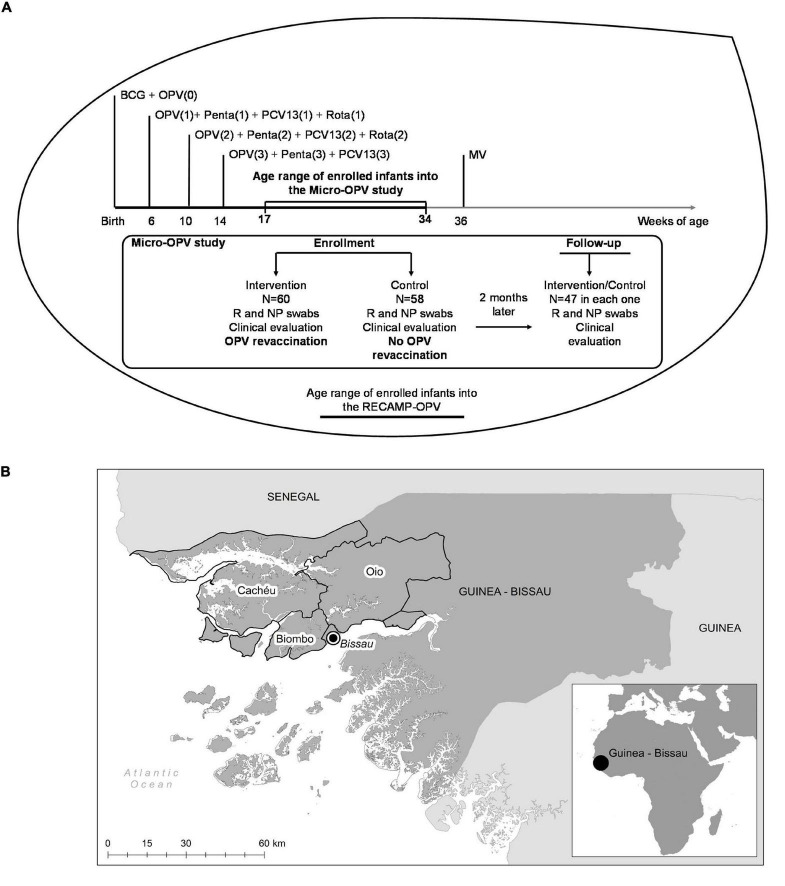
Experimental design and settings of the Micro-oral polio vaccine (OPV) study. **(A)** The Micro-OPV is a cohort study within a large cluster randomized clinical trial, the RECAMP-OPV. At enrollment, infants were clinically evaluated, and submitted to the collection of biological samples and, infants enrolled into the Intervention clusters were revaccinated with OPV, whilst infants enrolled into the Control clusters received no OPV revaccination. Two months later, 47 infants in each group were clinically evaluated and submitted to a new collection of biological samples. Vaccines such as Bacillus Calmette-Guérin (BCG), OPV, Pentavalent (DPT, HBV, Hib), PCV-13, Rotavirus, and measles vaccine (MV) are given as indicated as part of the national routine immunization plan. **(B)** Guinea-Bissau is on the west coast of Sub-Saharan Africa, as shown in the small picture at the bottom right corner. The villages participating in the Micro-OPV study, located in Cacheu, Biombo, and Oio highlighted regions, were within 2 h-drive from the capital Bissau.

All infants aged 17–34 weeks recruited to the RECAMP-OPV trial (Varma et al., 2019) (NCT03460002 on www.clinicaltrials.gov), and living in village clusters distant 2 h-drive from the capital Bissau were eligible to participate in the Micro-OPV cohort study (see the experimental design of Micro-OPV study in Figure 1A and the Guinea–Bissau map with the covering area of the Micro-OPV study in Figure 1B). This age range was planned because infants at those ages have already been eligible for a set of vaccines as part of the routine immunization plan, except MV (World Health Organisation [WHO], 2021a; Figure 1A). Area delimitation was due to logistic reasons concerning the transportation and preservation of biological samples, which are further detailed below. Biosampling took place from August 2018 to December 2018.

In RECAMP-OPV clinical trial, clusters were randomly classified 1:1 as Intervention clusters (with infants revaccinated with an extra dose of OPV in addition to those administrated according to routine immunization plan) or Control clusters (whose infants received no additional OPV) based on externally generated random numbers. All clusters received the same number of visits, at baseline and 2 months post-baseline. Due to the randomization design, recruited infants shared similar major microbiome determinants, such as, *at term* gestational age, vaginal delivery, and feeding progression. Given the exploratory nature of the Micro-OPV study, we conveniently planned to recruit 60 infants from each cluster to participate. At baseline and 2 months after baseline, in addition to the main trial procedures required by the RECAMP-OPV, including the administration of an extra dose of OPV to infants from the Intervention clusters at baseline, fecal and nasopharyngeal biological content was collected, and clinical data registered. A total of 47 participants out of 60 revaccinated with OPV and 47 participants out of 58 not revaccinated completed the 2 months follow-up in the Micro-OPV study.

Study villages are agricultural-based, served by well water and pit-latrines, and just 30–50% of them have access to electricity supply. Households live very close to each other, often sharing the same kitchen space and kitchenware, and people of the same household share the same food container. Also, people live very close to farm animals (chicken, pig, goat, and cow) and pets, such as dogs and cats.

### Procedures in the field at enrollment and follow-up under the Micro-OPV study

Procedures in the field under the Micro-OPV study were carried out in the rainy season (August to December) in 2018. After assurance of all ethical aspects described below, parents or legal guardians of selected infants attended a questionnaire-based interview at both time-points. All interviews were conducted in Créole, the *lingua-franca*, by the same BHP field worker under the researchers’ supervision. Information on major microbiome determinants and possible cofounders of the effect of OPV revaccination, such as housing conditions, mother’s health during pregnancy and delivery, and infant’s health since delivery to present, including delivery conditions, feeding history, previous infections, hospitalizations, and use of medicines were recorded at enrollment. Feeding progression, health problems, hospitalizations, and use of medicines since the enrollment were registered at follow-up.

After the interviews, infants were submitted to a clinical evaluation and measurement of axillary temperature. Anthropometric data [weight, height, and mid-upper-arm-circumference (MUAC)] were also noted. No infant met exclusion criteria at enrollment, namely, fever defined as an axillary temperature ≥38°C, MUAC < 110 mm in an infant older than 6 months or previous history of allergic reaction due to vaccination.

Nasopharyngeal (NP) samples and fecal (F) samples were collected from all participants at enrollment and 2 months later at follow-up using ultra minitip flexible and regular flocked swabs (FLOQSWABS^®^, Copan, Brescia, Italy), respectively. Immediately after the collection procedure, swabs were immersed in 1 ml of DNA/RNA Shield™ (Zymo Research, Irvine, CA, USA) in 15 ml Falcon tubes and kept cold until laboratory processing. At enrollment, after sampling procedures, one OPV-dose (two drops) was administered to infants belonging to the Intervention clusters, by the experienced BHP nurse. Infants belonging to the Control clusters received no intervention. No additional OPV dose was administrated between the two time-points to any of the participants.

The raw material was transferred from Falcon tubes to 2 ml Cryotubes in the laboratory facility of the Guinea-Bissau National Institute of Health in sterile conditions, kept at −20°C for 1 month in the BHP premises, transported to the Institute of Hygiene and Tropical Medicine, Universidade NOVA de Lisboa, Portugal (IHMT-NOVA) in dry ice and kept at −80°C until DNA extraction.

### DNA extraction

DNA was extracted from 500 μl of each raw sample through ZymoBIOMICS™ DNA Miniprep Kit (cat. no°.: D4300, Zymo Research, Irvine, CA, USA) following manufacturer’s orientations. Mechanical lysis was standardized through a ZymoBIOMICS™ Microbial Community Standard II (Log Distribution) (cat. n°.: D6310, Zymo Research, Irvine, CA, USA) using a Mini-BeadBeater device (model EUR16, BIOSPEC Products, Inc., Bartlesville, OK, USA). All DNA samples presented A_260_/A_280_ ratio >1.8 measured by a NanoDrop™ 1000 Spectrophotometer device (Thermo Fischer Scientific, Waltham, MA, USA) and 100 ng of DNA per sample, measured in a Qubit Fluorometer (Thermo Fischer Scientific, Waltham, MA, USA), were used to prepare the library.

### 16S rRNA amplicon deep sequencing

Paired (F and NP) extracted DNA samples of 94 participants who completed the 2 months of follow-up (47 OPV revaccinated infants and 47 controls) were deeply characterized by 16S rRNA amplicon sequencing to identify different changes in bacterial diversity and composition of gut (G) and upper respiratory (R) bacterial microbiotas over time. For that, paired-ended amplicon deep sequencing of V3 and V4 variable regions of the gene that codifies the 16S rRNA was performed using previously evaluated primers (341F: 5′-CCTACGGGNGGCWGCAG-3′; 805R: 5′-GACTACHVGGGTATCTAATCC-3′), preceded by heterogeneity spacers (Klindworth et al., 2013). Library construction and sequencing were performed on an Illumina MiSeq instrument (Illumina Inc., San Diego, CA, USA), using a 600 cycle V3 kit.

### Demultiplexing and taxonomic assignment

Demultiplexing was done with the *bcl2fastq* Conversion Software (Illumina Inc., San Diego, CA, USA). Cutadapt (version 2.3) was used to trim off heterogeneity spacers and primers at an error rate of 8% (allowing for one mismatch per primer) (Martin, 2011). The resulting sequences were quality-filtered, truncated, denoised, merged, and chimera-filtered through the DADA2 pipeline (version 1.12.1). High-resolution amplicon sequence variants (ASVs) were inferred from trimmed reads per run with default settings, except for truncation lengths (forward reads: 270 bp, reverse reads: 210 bp) (Callahan et al., 2016). Consensus removal of chimeras was performed on data from all runs. Samples with a read count <10,000 after quality filtering were re-sequenced. Taxonomic assignment of the resulting ASVs was done by means of dada2’s *assignTaxonomy()* and *addSpecies()* functions with the Silva reference database and species-level training set (version 132), respectively (Callahan, 2018). Subsequently, the ASV count table and the taxonomic table were integrated with the R package phyloseq (McMurdie and Holmes, 2013) to perform ecological and statistical analyses of microbiota data.

### Decontamination

The data set was split into F and NP samples and the R package *decontam* was used to identify and remove contaminants (Davis et al., 2018). For this, controls used in DNA extraction, the DNA/RNA Shield™ (Zymo Research, Irvine, CA, USA) solution and the controls from PCR were used. The *decontam* method “either” was used in F and NP samples data set adjusted for each one (frequency threshold 0.075, prevalence threshold 0.5 and frequency threshold 0.05, prevalence threshold 0.5, respectively). After computational contaminant removal, read counts of re-sequenced samples were merged. From the F sample data set, 67 ASVs were identified as contaminants and removed. In addition, 40 ASVs classified as Archaea, Cyanobacteria, Planctomycetes, Chloroflexi, Rhizobiales, Rhodobacterales, or Rhodospirillales were removed manually, as our major interest was to observe the behavior of the bacterial microbiota of gut and upper respiratory tract. Moreover, 61 ASVs that were not classified to at least order-level were excluded. Twenty-seven ASVs from the NP sample data set that were identified as contaminants were excluded. In addition, 99 ASVs that were not classified to at least order-level were excluded, as well as 224 ASVs classified as Eukaryota, Archaea, Cyanobacteria, Planctomycetes, Chloroflexi, Deinococcus-Thermus, Kiritimatiellaeota, Rhizobiales, Rhodobacterales, Rhodospirillales, or Oceanospirillales. A number of 2,373 ASVs were obtained from the 94 F samples, while 770 ASVs were obtained from the 94 NP samples, both distributed by seven taxonomic ranks. The average read count per sample after all pre-processing steps was 37,897 (F samples) and 31,139 (NP samples).

### Statistical analyses

Statistical significance levels were set at <0.05 for all tests used in analyses of epidemiological or sequencing data unless otherwise stated.

#### Epidemiological data

General characteristics at the enrollment and follow-up were described by group allocation (Intervention group with OPV revaccinated infants or Control group with not revaccinated controls) as proportions, averages, and medians, as appropriate, in IBM Statistics SPSS 25th version program. Differences in quantitative variables between groups were tested using Student’s *t*-test or Mann–Whitney *U* test, while within-group differences from enrollment to follow-up were tested using paired Wilcoxon signed rank test. Differences in qualitative variables between groups were tested using the Chi-square test or the Exact Fisher test, while within-group differences over time were tested using the McNemar test. We did not correct for the cluster, i.e., infants were considered independent in the analysis, which would tend to exaggerate potential group differences.

#### Sequencing data

Statistical analyses of microbiota characteristics were performed in R (version 4.0.4) (R Core Team, 2021). Graphs were created with ggplot2 (Wickham, 2016). Indexes of Shannon diversity (H) and Inverse of Simpson (InvSimpson) were used to assess alpha-diversity, i.e., differences in within-sample richness and evenness of microbiotas. Shannon diversity is a measure of alpha-diversity combining richness and evenness, where higher values indicate a high number of different taxonomic groups homogeneously distributed in a community. InvSimpson is a measure of dominance or a complementary measure of evenness, where higher values indicate homogeneous proportions of taxonomic groups and low dominance levels. They were compared between groups at enrollment using Mann–Whitney *U* test and within-groups over time using paired Wilcoxon signed rank test.

A core microbiota was defined as the subset of ASVs with at least five reads in at least two samples. The count data was then Hellinger transformed, that is calculation of sample-wise proportions and subsequent square root transformation, to account for differences in samples’ library sizes and zero inflation. Principal co-ordinates analysis (PCoA) based on the Bray–Curtis distance for visualization of differences in the bacterial composition between OPV revaccinated and controls and between time-points was performed. Differences in bacterial composition between groups and time points were tested with permutational multivariate analysis of variance using distance matrices (PERMANOVA) with the *adonis()* function from the vegan package (version 2.5-5) (Oksanen et al., 2019). As a pre-requisite, group homogeneity of variances was tested by implementing the *betadisper()* function. Additionally, pairwise comparisons of within- and between-group Bray-Curtis dissimilarities of all group-time point combinations were made using a pairwise Wilcoxon rank sum test with continuity correction and *p*-values adjusted with Benjamini–Hochberg correction. Taxonomic heat trees based on relative abundance up to genus level were generated with the package metacoder (Foster et al., 2017) and used to visualize differences in relative abundances between enrollment and follow-up within OPV revaccinated and controls. Differentially abundant taxa between enrollment and follow-up within groups were identified by linear discriminant analysis (LDA) effect size (LEfSe) on count data normalized to the sum of 1e + 06 with an LDA cut-off of 4 (package microbiomeMarker) (Cao, 2020). Taxa identified more than three times in at least 5% of the samples were considered in the analysis. A high LDA score (log10) indicates a high effect size of the respective taxon for explaining group difference. LEfSe allows the identification of differentially abundant taxa on several taxonomic levels (here kingdom to species) by accounting for the hierarchical structure of bacterial phylogeny.

## Results

The following results gather data from 47 participants in each group who completed the follow-up, according to the experimental design of the Micro-OPV study shown in Figure 1A.

### OPV revaccinated and control infants shared similar epidemiological profiles and major microbiome determinants

No major differences in participant profiles were observed between the 47 (60% female) OPV revaccinated infants (participants in the Intervention group) and the 47 (49% female) controls (participants in the Control group) at enrollment (Table 1). Although a significantly higher mean length was observed for OPV-revaccinated infants at enrollment, it may reflect the slightly higher age of the infants in this group and, possibly missed length measures for six participants in the Control group and for two OPV-revaccinated participants. This difference was no longer observed at follow-up when complete data were recorded from all infants. No major differences in clinical conditions anticipated to affect the microbiome at the time of sampling and during the 2 months follow-up were observed between groups (Tables 1, 2).

**TABLE 1:** Participant profiles at the enrollment and follow-up.

Characteristics	Enrollment			Follow-up		
	Intervention (*n* = 47)	Control (*n* = 47)	*p*	Intervention (*n* = 47)	Control (*n* = 47)	*p*
**Gender^a^**						
Male	19 (40)	24 (51)	0.30			
Female	28 (60)	23 (49)				
Age (months)^b^	6.68/5.86–7.53	6.09/5.10–7.24	0.12	8.65/7.66–9.44	8.13/7.01–9.18	0.10
**Anthropometric data^c^**						
Weight (kg)	7.4/4.7–10/1.1	7.1/4.5–10/1.1	0.27	7.9/5.4–11/1.2	7.5/4.8–11.2/1.3	0.14
Length* (cm)	67/58–74/3	66/61–72/3	**0.01**	69/59–75/3	68/61–74/3	0.27
(MUAC) (mm)	135/108–160/11	135/108–164/12	0.99	139/112–170/12	136/112–180/14	0.34
**Clinical signs at sample collection**						
Axillary temperature** (°C)^c^	36.3/35.5–37.2/0.34	36.3/35.1–37.5/0.45	0.70	36.0/35.5–36.8/0.34	36.2/35.0–37.4/0.48	**0.03**
Urticaria^a^	4 (9)	5 (11)	0.73	0	0	
Cough^a^	11 (23)	15 (32)	0.36	10 (21)	17 (36)	0.11
Running nose—common cold^a^	27 (57)	26 (55)	0.84	23 (49)	21 (45)	0.68
Diarrhea^a^	3 (6)	5 (11)	0.56	7 (15)	5 (11)	0.54

^*a*^Number (%).

^*b*^Median/Interquartile range.

^*c*^Average, minimum-maximum, standard deviation.

*Two missing values in Intervention group and six missing values in Control group at enrollment.

**Eight missing values in Intervention group at follow-up.

*p P*-value; differences between Intervention and Control groups were tested using Student’s *t*-test or Mann–Whitney *U* test for quantitative data and Chi-square test or Exact Fisher test for qualitative data. Significant differences are displayed in bold.

**TABLE 2:** Clinical outcomes developed from enrollment to follow-up.

Characteristics		Intervention (*n* = 47)	Control (*n* = 47)	*p*
**Acute respiratory infection^a^**				
Cough		19 (40)	26 (55)	0.15
Shortness of breath episode		16 (34)	19 (40)	0.52
Any episode fast breathing		11 (23)	8 (17)	0.44
Any episode fever		21 (45)	20 (43)	0.84
**Acute gastroenteritis**				
Diarrhea^a^		16 (34)	16 (34)	1.00
Diarrhea episodes^b^		1.25/1–3/0.6	1.88/1–7/1.5	0.14
Feces aspects^a^				
	Watery	16 (100)	14 (87)	
	With blood	0	1 (6)	0.34
	With mucus	0	1 (6)	
Malaria episode^a^		2 (4)	2 (4)	1.00
Hospitalization^a^		2 (4)	1 (2)	0.56
Use of medicines^a^		22 (47)	18 (38)	0.40
Paracetamol		17 (36)	12 (25)	0.26
Antibiotics		7 (15)	3 (6)	0.18
	Amoxicillin	5 (71)	2 (67)	0.88
	Cotrimoxazole	1 (14)	1 (33)	0.49
	Ceftriaxone + Amoxicillin + Azithromycin	1 (14)	0	–
	Antimalarial coartem	1 (2)	0	–

^*a*^Number (%).

^*b*^Average, minimum-maximum, standard deviation.

Participant profiles after excluding infants that used antibiotics during follow-up (seven OPV revaccinated infants and three controls) were similar to that shown for the 47 infants in each group (Supplementary Tables 6, 7).

Infants in both groups were similar in characteristics of major microbiome determinants at enrollment, such as, environmental, birth, diet, and clinical factors that influence the microbiome development (see Supplementary Tables 1–4). Supplementary Table 3 describes, specifically, the feeding history of participants at enrollment and follow-up. No significant differences were observed between groups. Breastfeeding coverage was very high among all participants at both time-points. The same was observed regarding porridge feeding and water administration, including median number of doses administrated per week. Regarding another sources of nutrients, fruits were introduced at follow-up for both groups of infants, however, to a lower extent. Significant differences were observed regarding a lower number of residents per house younger than 15 years old (5 vs. 6, *p* = 0.02) in the group with OPV revaccinated infants, together with a relatively higher use of tap water (5 vs. 0, *p* = 0.03) and charcoal to cook (6 vs. 0, *p* = 0.03) in this same group, although most infants in both groups lived in households which used well water and wood to cook. All deliveries in both groups came from *at term* pregnancies of newborns born vaginally and almost 50% at home. Few mothers and infants in both groups had been tested for human immunodeficiency virus (HIV), and no positive cases had been detected. Chemoprophylaxis to malaria in pregnancy was taken by almost 80% of mothers and few of them reported malaria in pregnancy. Most infants were exclusively breastfed up to the 5th month of life, and then started to receive porridge and water. Few infants had malaria before enrollment, while a higher proportion had already experienced diarrhea episodes. More than 50% of infants in both groups had used medicines up to 1 month before enrollment, mainly Paracetamol and equally few infants in both groups had used antibiotics previously, mainly Amoxicillin. No significant differences were observed regarding the proportion of infants in both groups with complete doses of recommended vaccines according to routine immunization schedule until 14 weeks of age (World Health Organisation [WHO], 2021a).

### OPV revaccinated and control infants exhibited similar changes in alpha-diversity over time for both microbiotas

**FIGURE 2:**
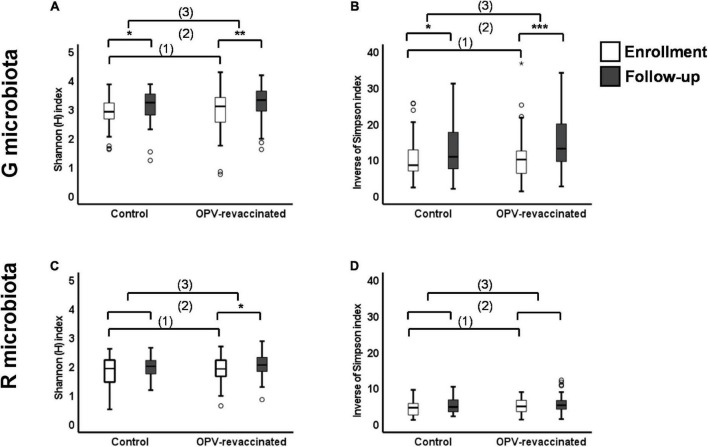
Changes in alpha-diversity over time in G and R microbiotas of infants. Shannon (H) index **(A,C)** and Inverse of Simpson index **(B,D)** values were compared: (1) between Control and oral polio vaccine (OPV) revaccinated infants at enrollment without significant differences (*p* > 0.05) for any comparisons, (2) within each group over time with significant differences for G microbiota in both groups [**(A)** Control *p* = 0.038 and OPV revaccinated *p* = 0.001; **(B)** Control *p* = 0.047 and OPV revaccinated *p* < 0.001] and R microbiota in OPV revaccinated [**(C)** OPV revaccinated *p* = 0.01], and (3) the magnitude of changes over time was compared between groups without significant differences for any comparisons. *p < 0.05; **p < 0.01; ***p < 0.001; the absence of asterisks for any comparisons indicates that there are no significant differences. Mann–Whitney *U* test was used for comparisons between groups and paired Wilcoxon signed rank test was used for comparisons within groups over time.

The distribution of Shannon (H) and Inverse of Simpson (InvSimpson) indexes in both groups at enrollment and follow-up is shown in Figure 2.

First, we showed that infants in both groups presented similar indexes for both microbiotas at enrollment as observed in Figures 2A,B (for G microbiota) and Figures 2C,D (for R microbiota), evidencing similar exposition to major microbiomes determinants between groups, as previously shown. Then, both indexes significantly increased from enrollment to follow-up in G microbiota of OPV revaccinated infants (H *p* = 0.001, InvSimpson *p* < 0.001) and controls (H *p* = 0.03, InvSimpson *p* = 0.03), whereas in R microbiota, the H index only significantly increased in OPV revaccinated infants (*p* = 0.011). Changes in InvSimpson index in R microbiota were not significant for both groups in the same period.

Next, we compared the magnitude of changes over time for both microbiotas in each group to test whether they might still be greater in OPV revaccinated infants due to revaccination. A similar magnitude of changes in alpha-diversity over time [comparison (3) in Figure 2] was found in G (Figures 2A,B) and R microbiotas (Figures 2C,D) in infants from both groups.

Similar results were obtained after excluding infants in both groups who used antibiotics during follow-up period (data not shown).

In summary, alpha-diversity for both microbiotas were similar between groups at enrollment, i.e., before infants in the Intervention group have received OPV revaccination. Two months later, we observed that alpha-diversity increased similarly in both groups over time, despite infants in the Intervention group have received the OPV boost.

### OPV revaccinated infants exhibited larger changes over time in bacterial composition for both microbiotas than controls

Visualization of differences in bacterial composition between groups and time-points for G and R microbiota samples were performed using PCoA based on Bray–Curtis dissimilarities (Supplementary Figures 1A,B). After ensuring homogeneity in whitin-group variations by implementing the *betadisper()* function (ANOVA, *p* > 0.05), PERMANOVA analyses for G and R microbiotas showed that differences between bacterial composition were based on group and time point for G (*p* < 0.01) and R (*p* = 0.001) bacterial microbiotas. When analyses were deepened to identify whether differences were attributable to time point, group or an interaction of both, we identified that differences for G bacterial microbiota were more attributable to time point (*p* < 0.01) than group (*p* > 0.05) or an interaction of both (*p* > 0.05), whilst for R bacterial microbiota differences were attributable to time-point (*p* < 0.05) and group (*p* = 0.001) but not due to an interaction of both (*p* > 0.05).

To assess differences in bacterial composition for both microbiotas over time and to test if they were larger in OPV revaccinated infants than controls, firstly, data were split according to time-point and G and R samples from OPV revaccinated and controls at enrollment were analyzed. After Hellinger transformation of data and ensuring homogeneity in within-group variations by implementing the *betadisper()* function (ANOVA, *p* > 0.05), PERMANOVA analyses showed no differences between groups regarding bacterial communities in G (*p* > 0.05) and R (*p* > 0.05) microbiotas at enrollment, i.e., at baseline, before infants in the Intervention group being revaccinated with an extra-dose of OPV. Next, we split up phyloseq object by group and tested if there was a difference in within-group bacterial composition between samples at enrollment and samples collected 2 months later. After Hellinger transformation of data and testing for homogeneity in whitin-group variation by implementing the *betadisper()* function (ANOVA, *p* > 0.05), PERMANOVA analyses showed significant differences over time in bacterial composition in R microbiota samples of OPV revaccinated infants (*p* < 0.05) and controls (*p* = 0.01), indicating changes over time in R bacterial microbiota samples in both groups. Using the same methodology, PERMANOVA analyses showed that the bacterial composition of G microbiota samples of controls was different between time points (*p* < 0.05). Heterogeneity in within-group variation over time in G microbiota samples of OPV revaccinated infants avoided PERMANOVA analyses to verify differences in bacterial composition over time. This fact suggests that changes over time had occurred in G microbiota of OPV revaccinated infants, at least in some samples.

**FIGURE 3:**
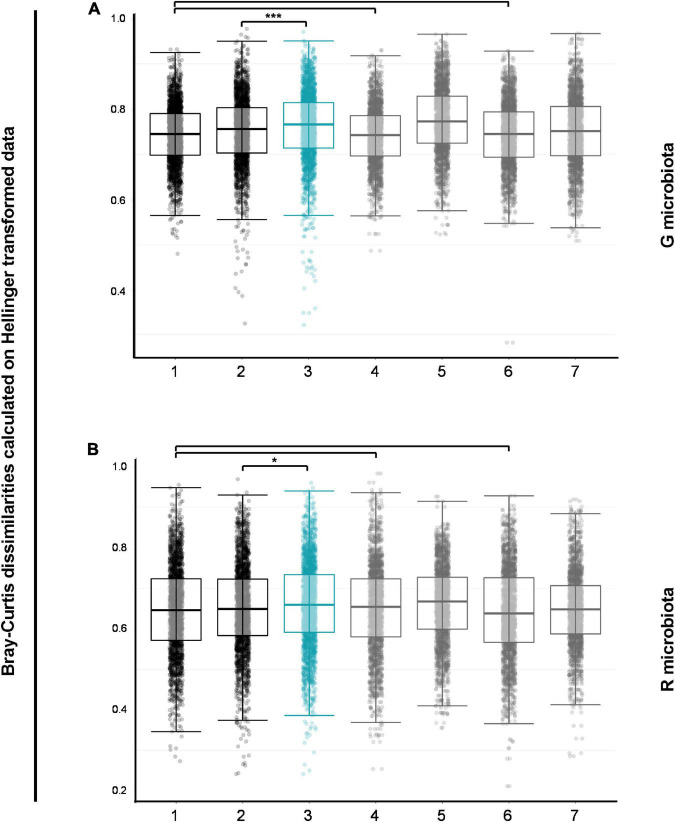
Pairwise comparisons of Bray–Curtis dissimilarities for G and R microbial communities. Bacterial communities in G **(A)** and R **(B)** microbiotas in both groups at enrollment (first black boxes) were similar to bacterial communities within the two groups at enrollment (first and third gray boxes). Control group was used as a reference for changes in oral polio vaccine (OPV) revaccinated over time and bacterial communities in G **(A)** and R **(B)** microbiotas changed significantly more over time in OPV revaccinated than in Control group (blue boxes *versus* second black boxes, respectively, *p_*adjusted*_* < 0.001 for G microbiota and *p_*adjusted*_ = 0.018 for R microbiota). *p < 0.05; **p < 0.01; ***p < 0.001; the absence of asterisks for any comparisons indicates that there are no significant differences. Pairwise Wilcoxon rank sum test with continuity correction and *p*-values adjusted with Benjamini–Hochberg correction for pairwise comparisons of within- and between-group Bray–Curtis dissimilarities of all group-time point combinations. 1—OPV revaccinated_Enrollment_ vs. Control_Enrollment_; 2—Control_Enrollment_ vs. Control_*Follow–up*_; 3—OPV revaccinated_Enrollment_ vs. OPV revaccinated_Follow–up_; 4—Whithin OPV revaccinated_Enrollment_; 5—Whithin OPV revaccinated_Follow–up_; 6—Whithin Control_Enrollment_; 7—Whithin Control_Follow–up_.

To assess whether changes over time in bacterial composition were greater in OPV revaccinated than controls, we carried out pairwise comparisons of within- and between-group Bray–Curtis dissimilarities on Hellinger transformed data. Data for G and R microbiotas are shown in Figures 3A,B, respectively. First, we observed that bacterial communities in G microbiota in both groups at enrollment (first black box, Figure 3A) were no more different from each other than within the two groups at enrollment [first (*p_*adjsted*_* = 0.524) and third gray boxes (*p_*adjusted*_* = 0.540), Figure 3A]. The same was found for bacterial communities in R microbiota [first and third gray boxes (*p_*adjusted*_* = 0.199), Figure 3B]. Then, the Control group was used as a reference for changes in OPV revaccinated infants over time, showing that bacterial communities in G microbiota changed significantly more over time in OPV revaccinated infants than controls (blue box vs. second black box, respectively, *p_*adjusted*_* < 0.001, Figure 3A). Results for R microbiota samples were similar, also showing a significantly larger change over time in OPV revaccinated infants than controls (blue box *vs.* second black box, respectively, *p_*adjusted*_* = 0.018, Figure 3B).

In summary, results suggest that changes over time in the composition of G and R bacterial microbiotas occurred in both groups and were greater in OPV revaccinated infants. The large within-group variation found in G microbiota samples in the group of OPV revaccinated infants at follow-up may suggest favorable growth of specific taxa in certain samples. Similar results were found after excluding antibiotic users in both groups (data not shown).

### Taxonomic composition for both microbiotas changed differently over time in OPV revaccinated infants than controls

Differences in the relative abundance of the 50 most abundant genera and higher taxonomic levels for both microbiotas and groups over time were visualized in differential taxonomic heat trees shown in Supplementary Figures 2A–D, fully described in the same Supplementary material.

**FIGURE 4:**
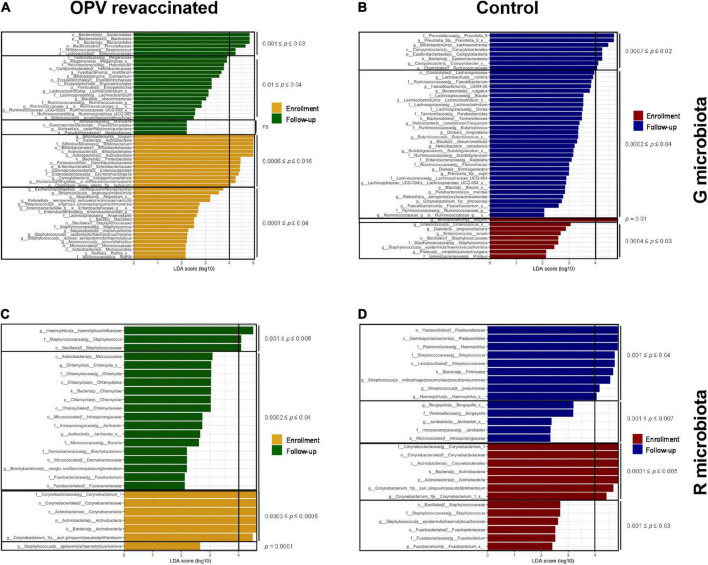
LefSe analysis on species level and higher hierarchical groups identified taxa groups that significantly changed over time. Taxa in yellow and dark red significantly decreased over time, whereas taxa in green and blue significantly increased over time in G and R microbiotas in oral polio vaccine (OPV) revaccinated [**(A,C)**, respectively], and Control [**(B,D)**, respectively]. Adjusted *p*-values are displayed separately for taxa with 0 ≤ LDA score (log10) < 4 and LDA score (log10) ≥ 4. Taxa with highest effect size [LDA score (log10) ≥ 4] are the most responsible for the significant difference over time in each group.

LEfSe analysis was used to identify taxa groups that significantly changed in both microbiotas and groups over time (LDA score ≥4). Figure 4A illustrates which taxa groups significantly decreased over time in OPV revaccinated infants (yellow bars), i.e., that were higher at enrollment, and which ones significantly increased over time (green bars). *B. longum*, *Escherichia*/*Shigella*, *Campylobacter*, and *Clostridium butyricum* are the taxa that most decreased in OPV revaccinated (range of *p*_*adjusted*_ values from 0.0006 to 0.016), whereas Prevotellaceae and *Streptococcus* were the ones that most increased over time (range of *p*_*adjusted*_ values from 0.001 to 0.03). A small number of taxa (dark red bars in Figure 4B) significantly decreased in G microbiota in the Control group, mainly *B. longum* (*p*_*adjusted*_ = 0.01), whereas a greater number of taxa significantly increased over time (blue bars in Figure 4B), mainly *Prevotella*_9, *Bifidobacterium kashiwanohense* and *Campylobacter* (range of *p*_*adjusted*_ values from 0.0007 to 0.02).

*Corynebacterium*_1 was the taxon that most decreased in R microbiota in OPV revaccinated (yellow bars in Figure 4C, range of *p*_*adjusted*_ values from 0.0003 to 0.0006), whereas *Haemophilus* (*p*_*adjusted*_ = 0.006) and *Staphylococcus* (*p*_*adjusted*_ = 0.002) were those that most increased over time (green bars in Figure 4C). *Corynebacterium*_1, *Staphylococcus*, and *Fusobacterium* were those that most decreased in the Control group, mainly the former (*p_*adjusted*_* = 0.0002, dark red bars in Figure 4D), whereas *Haemophilus* (*p_*adjusted*_* = 0.03) and *Streptococcus p_*adjusted*_* = 0.001) were the ones that most increased over time (blue lines in Figure 4D).

**FIGURE 5:**
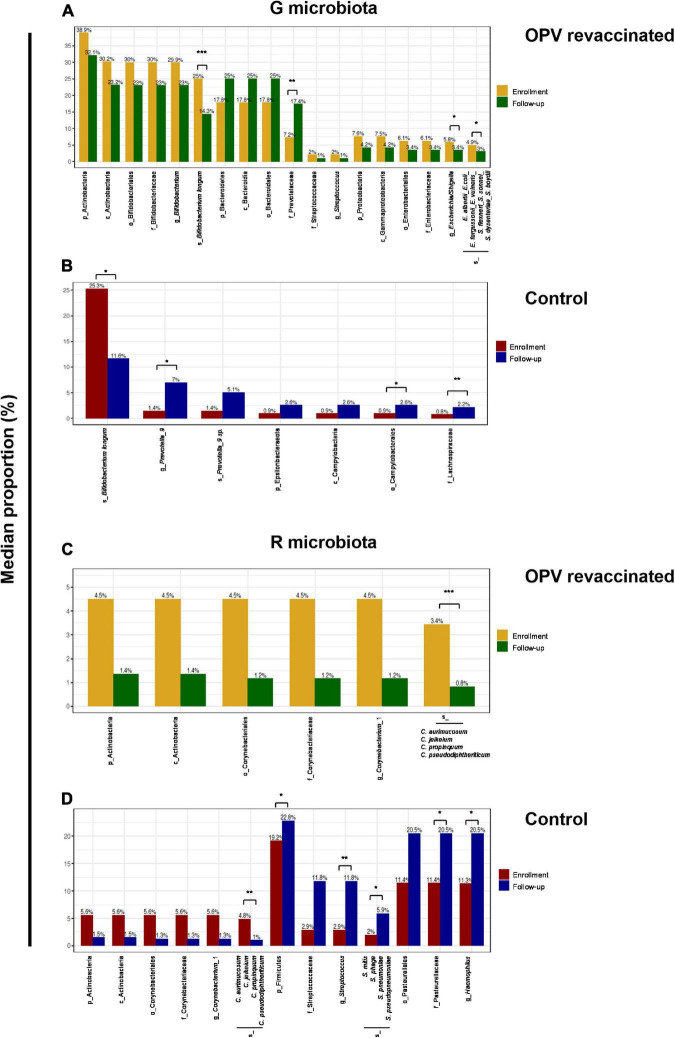
Linear discriminant analysis effect size (LefSe) identified taxa with relative abundance greater than 1% that significantly changed over time. Differences in median proportion between taxa with a relative abundance greater than 1% that significantly changed from enrollment to follow-up in G and R microbiotas in oral polio vaccine (OPV) revaccinated infants **(A,C)**, respectively and Control **(B,D)**, respectively are shown. Median proportion of each taxon at both time points are displayed on the top of the columns. All differences over time shown in the graphics are significant. Adjusted *p*-values are displayed for those taxa mentioned in the text. *p < 0.05; **p < 0.01; ***p < 0.001.

When comparing changes over time among taxa with relative abundance greater than 1%, we can observe more taxa groups in OPV revaccinated (Figure 5A) than in controls (Figure 5B) that significantly changed in G microbiota. The opposite occurred in R microbiota, for OPV revaccinated (Figure 5C) and controls (Figure 5D). A significant decrease in the median proportion of *B. longum* over time was observed both in OPV revaccinated (25–14.3%, *p*_*adjusted*_ = 0.0006) and controls (25.3–11.6%, *p*_*adjusted*_ = 0.01) (Figure 5A). A significant increase in the median proportion of Prevotellaceae (7.2–17.4%, *p*_*adjusted*_ = 0.005) family was observed in OPV revaccinated infants (Figure 5A), whereas a significant increase in the median proportion of *Prevotella*_9 genus was observed in the Control group (1.4–7.0%, *p*_*adjusted*_ = 0.02) (Figure 5B). Additionally, a significant decrease in *Escherichia*/*Shigella* (5.8–3.4%, *p*_*adjusted*_ = 0.01) was observed within OPV revaccinated group over time (Figure 5A), with a significant decrease of an undetermined *Escherichia/Shigella* species (4.9–3%, *p*_*adjusted*_ = 0.02). On the other hand, the median proportion of Campylobacterales (0.9–2.6%, *p*_*adjusted*_ = 0.03) and Lachnospiraceae (0.8–2.2%, *p*_*adjusted*_ = 0.007) significantly increased over time in G microbiota in the Control group (Figure 5B). The main change in R microbiota of the OPV revaccinated infants over time (Figure 5C) was the significant reduction in the median proportion of a *Corynebacterium_1* species (3.4% to 0.8%, *p*_*adjusted*_ = 0.0006). The same was also observed in the Control group (4.8–1%, *p*_*adjusted*_ = 0.005) (Figure 5D), but simultaneously with an increase in the median proportion of other several taxa groups (Figure 5D), such as Firmicutes (from 19.2–22.8%, *p*_adjusted_ = 0.04), *Streptococcus* genus (2.9–11.8%, *p*_adjusted_ = 0.001) and an undetermined *Streptococcus species* (2–5.9%, *p*_adjusted_ = 0.01), followed by the increase in Pasteurellaceae family (11.4–20.5%, *p*_adjusted_ = 0.03), including organisms from *Haemophilus* genus (11.3–20.5%, *p*_adjusted_ = 0.03).

In summary, taxonomic composition of G and R microbiotas changed differently over time in OPV revaccinated infants vs. controls. A more proportional alternance over time between the most abundant commensals (*B. longum* and Prevotellaceae) was observed in G microbiota of OPV revaccinated infants. Also, it was observed a more abundant and diversified community of Prevotellaceae in G microbiota of OPV revaccinated infants, followed by a reduction in the median proportion of *Escherichia*/*Shigella* and limited increase in the median proportion of Campylobacterales. In R microbiota, it was observed a limited increase in the median proportion of *Streptococcus* and *Haemophilus* in OPV revaccinated infants, whereas in controls these taxa were present among those with relative abundance greater than 1% at follow-up. Similar results were found after excluding antibiotic users in both groups (data not shown).

## Discussion

This is the first study that used 16S rRNA deep sequence analysis of gut and upper respiratory bacterial content to show changes in the composition of gut and upper respiratory microbiomes after OPV revaccination.

Strengths of the study includes the cluster-randomized set-up resulting in similar epidemiological profiles and characteristics of major microbiome determinants in both groups at enrollment. This allowed us to provide unbiased effects of OPV revaccination on the selected microbiomes. High-quality sequencing results reflect the good strategies adopted for sequencing, as well as for sample collection, storage, and DNA extraction. A 2-month follow-up period was planned to maximize detection of changes in both microbiomes after OPV revaccination, as in that period, replication of the live-attenuated PV and an intense activity of host immune system due to revaccination are ongoing (Connor et al., 2021). Also, a longer observation period for weaning age infants could dilute the results. Main weaknesses found in the present study were the intrinsic limitation of 16S rRNA gene deep sequencing analysis for species discrimination and the lack of functional analysis of microbiome content. As the clustering was not considered in the statistical analyses of epidemiological data and due to small number of participants in the Micro-OPV study, there was no power to make any conclusions regarding clinical protection to unrelated pathogens conferred by OPV-associated changes in bacterial microbiota.

Regarding gut microbiota composition, our results are in line with the successive colonization events of the intestinal tract, that are described (Moore and Townsend, 2019; Dizzell et al., 2021) for *at term* vaginally born infants, exclusively breastfed until 6 months of life and on a recent weaning process with the introduction of starches through porridges, such as participating infants in the Micro-OPV study. *B. longum* was the major representative of the Actinobacteria phylum in G microbiotas for both groups at enrollment, corroborating literature data that point to *B. longum*, *Bifidobacterium breve* and *Bifidobacterium bifidum* as the dominant bacteria in G microbiota of breastfed infants, as these commensal bacteria are the main metabolizers of human milk oligosaccharides (O’Callaghan and van Sinderen, 2016; Le Doare et al., 2018). A high breastfeeding coverage, given as the only nutrient source until 6 months of age, was observed among the participating infants. At follow-up, we found a significant decrease in Actinobacteria and increase in Bacteroidetes for both groups. A higher porridge coverage at follow-up in both groups, as shown in Supplementary Table 3, explains this finding and corroborates literature data which point to an increase in Bacteroidetes after introduction of more solid foods, mainly starches (Houghteling and Walker, 2015; Moore and Townsend, 2019). Also, the marked abundance of Bacteroidetes is in accordance with what has been previously described for infants living in rural African villages (De Filippo et al., 2010). Despite no differences between the OPV revaccinated infants and the control infants regarding feeding history and progression, a more diverse and pronounced increase in relative abundance of the Prevotellaceae family, comprising several *Prevotella* genera, was observed in OPV revaccinated infants, whereas a less expressive increase of this taxon, restricted to *Prevotella*_9 genus occurred in controls. Even though a similar greater proportion of infants in both groups at enrollment were fed with porridge and this number increased similarly in both groups at follow-up, a higher diversity in Bacteroidetes was observed in OPV revaccinated infants at follow-up, followed by a more proportional alternance over time between the median proportions of Actinobacteria and Bacteroidetes compared to Control group. A decrease in potentially pathogenic/opportunistic organisms of Proteobacteria, such as *Escherichia/Shigella*, is expected for weaning infants and was observed in OPV revaccinated infants over time, followed by a decrease in the relative abundance of *Streptococcus*. The same decrease was not observed in the Control group and, indeed, an increase over time in Campylobacterales was observed together with a more pronounced increase in families such as Lachnospiraceae and Ruminococcaceae known to codominate the fecal bacteria of healthy adults (Ishiguro et al., 2018).

These results reveal a potential OPV beneficial effect associated with a greater diversity and abundance of important gut commensal bacteria, such as those from the Prevotellaceae family, known producers of short-chain fatty acids (SCFA), and decrease in potentially pathogenic/opportunistic taxa. SCFA, such as acetate, propionate and butyrate, are the final products from the metabolism of non-digestible dietary fibers and play a role in the modulation of gut immune system (Flint et al., 2014; Deleu et al., 2021). They are involved in epigenetic regulation of innate immune cells and possibly, in the developmental process of innate memory (Danelishvili et al., 2019), strengthening the hypothesis of trained immunity mechanisms behind NSE of OPV and due to changes in bacteria microbiota. In contrast, control infants that did not receive OPV revaccination developed a more imbalanced gut bacterial microbiota composition, with relatively more potential opportunistic bacteria and with some of them expected to be present in older stages of life.

We cannot unambiguously determine the exact species of *Escherichia*/*Shigella* that was reduced in OPV revaccinated infants, since 16S rRNA sequencing analysis has limitations for species level discrimination. Hence, we cannot confirm a direct OPV effect against pathogenic bacteria in this taxon.

R microbiota results are also in line with what has been previously described for nasopharyngeal colonization (Lanaspa et al., 2017). We observed in OPV revaccinated that significant changes in most abundant taxa were restricted to a decrease in the median proportion of an opportunistic *Corynebacterium*_1 species after OPV revaccination. In the opposite, several taxa increased in the Control group at follow-up, including potential causative genera of upper and lower respiratory infections, such as *Streptococcus* and *Haemophilus.* As observed above for changes in G microbiota after OPV revaccination, changes in R microbiota also suggest that OPV revaccination was associated with decrease in some potential opportunistic species under the *Corynebacterium* genus, without changing the dominance of this taxon, which is expected to be the dominant one in breastfeed infants. Also, our results suggest that OPV revaccination may limit the increase in potential pathogenic organisms of *Streptococcus* and *Haemophilus* genera, that are expected to colonize the oropharynx in later stages of life.

Here, we presented changes in gut and upper respiratory microbiomes associated with OPV revaccination, characterized by the development of a healthier microbiome composition. Our study was too small to study the clinical implications, so, it remains to be studied whether the OPV-associated changes in bacterial microbiota of gut and upper respiratory tract are associated with beneficial NSE conferred by OPV revaccination. A similar experimental set up including more infants and detailed information on etiological diagnosis of specific clinical endpoints, species level differentiation at microbiota analysis and functional analysis of microbiome content using metabolomics would be helpful to elucidate this question.

In conclusion, the Micro-OPV study showed that OPV revaccination vs. no revaccination was associated with changes in gut and upper respiratory bacterial microbiotas toward a healthier composition. This comprises a greater diversity and abundance of gut commensals potentially producers of immune modulatory molecules involved in trained immunity, such as, short-chain fatty acids, and a lower proportion of potential pathogenic/opportunistic genera for both microbiotas.

## Data availability statement

The datasets presented in this study can be found in online repositories. The names of the repository/repositories and accession number(s) can be found below: https://www.ncbi.nlm.nih.gov/, BioProject ID: PRJNA773649.

## Ethics statement

The studies involving human participants were reviewed and approved by Comité Nacional de tica em Saúde (CNES)-Guinea-Bissau. Written informed consent to participate in this study was provided by the participants’ legal guardian/next of kin.

## Author contributions

MMM, ABF, CSB, ML, and HS: conceptualization. MMM, ACI, MS, PSA, ABF, ML, and PA: methodology. MMM, ACI, MS, and PA: formal analysis. ABF, ML, and HS: funding acquisition. MMM, LMN, CC, ABF, and ML: investigation. MMM, ML, and PA: project administration. MMM, ML, HS, and PA: supervision. MMM and ACI: writing—original draft. LMN, MS, PSA, ABF, CSB, HS, and PA: Writing—review and editing. All authors have read and agreed to the published version of the manuscript.
