# Supplementary material for: Oral polio revaccination is associated with changes in gut and upper respiratory microbiomes of infants
Source: Front Microbiol. 2022 Oct 28;13:1016220. doi: 10.3389/fmicb.2022.1016220 (PMC9649904; doi:10.3389/fmicb.2022.1016220)
Supplement: Supplementary file 1 [file Data_Sheet_1.pdf]

Oral polio revaccination is associated with changes in gut and upper respiratory microbiomes of infants

Supplementary Figure 1. PCoA based on Bray-Curtis dissimilarities for visualization of differences in bacterial composition between groups and time points for G and R microbiota samples. Samples for (**A**) G and (**B**) R microbiotas are displayed according to different group and time point combinations. PERMANOVA analyses showed that samples from both groups exhibited similar bacterial composition at enrollment and identified significant differences in bacterial composition over time for G and R microbiota samples in the Control group (**A**) and (**B**), bottom right corner, respectively) and for R microbiota samples in OPV revaccinated (**B**), top left corner. Heterogeneity in within-group variation over time in G microbiota samples in OPV revaccinated avoided a valid PERMANOVA analyses.

Supplementary Figure 2. Taxonomic heat trees based on relative abundance up to genus level within-groups over time for G and R microbiotas. Differences in relative abundance between enrollment and follow-up of the 50 most abundant genera and above within-group are shown. Nodes represent each taxon and the lines between them show the hierarchical relationships between taxa. Node colors indicate the percent point difference between enrollment and follow-up, allowing visualization of the most abundant taxa that decreased from enrollment to follow-up (yellow and red dark) and increased in the same period (green and blue) in OPV revaccinated and Control, respectively, for G (**A**) and R (**C**) microbiotas in OPV revaccinated, and (**B**) G and (**D**) R microbiotas in Control. Node sizes (diameters) indicate the overall relative abundance of each taxon at both time points.

We observed in G microbiota of infants in both groups the marked overall relative abundance of clades that decreased from enrollment to follow-up comprising Actinobacteria to *Bifidobacterium* and Proteobacteria to *Escherichia*/*Shigella* for both groups (yellow nodes in **Figure 2A** and dark red nodes in **Figure 2B**), followed by Bacilli to *Streptococcus* and Epsilonbacteraeota to *Campylobacter* in OPV revaccinated (yellow nodes in **Figure 2A**) and, Firmicutes to *Megasphaera* and *Veillonella*, followed by Firmicutes to *Streptococcus* in the Control group (dark red nodes in **Figure 2B**). A decrease over time in *Prevotella* and *Bacteroides* genera was also observed in the Control group (dark red nodes in **Figure 2B**). Taxonomic groups that increased from enrollment to follow-up and presented a greater overall relative abundance in G microbiota were represented by Bacteroidetes to *Prevotella*_9, *Prevotella*, *Prevotella*_6 and *Prevotela*_2, Bacteroidetes to Bacteroides, Firmicutes to

Lachnospiraceae and Firmicutes to *Megasphaera* in OPV revaccinated infants (green nodes in **Figure 2A**), and by Bacteroidetes to *Prevotella*_9, almost the exclusive generum that increased over time in Prevotellaceae family, Clostridia to Lachnospiraceae, Coriobacteriia to *Collinsella* and Epsilonbacteraeota to *Campylobacter* in the Control group (blue nodes in **Figure 2B**). The percent point difference between enrollment and follow-up was larger in OPV revaccinated than controls, showing that the magnitude of changes over time in the relative abundance of the 50 most abundant genera and above in G microbiota was greater in OPV revaccinated infants.

We observed in R microbiota the marked overall relative abundance of clades that decreased from enrollment to follow-up comprising Proteobacteria to *Haemophilus* in OPV revaccinated (yellow nodes in **Figure 2C)** and Proteobacteria to *Moraxella* in the Control group (dark red nodes in **Figure 2D**). This was followed by Actinobacteria to *Corynebacterium*_1 and Camobacteriaceae to *Dolosigranulum* in both groups. In the same figures, the most abundant taxa groups that increased from enrollment to follow-up for both groups comprises Firmicutes to *Streptococcus*, followed by Bacteroidetes to *Ornithobacterium* in R microbiota in OPV revaccinated (green nodes in **Figure 2C**) and controls (blue nodes in **Figure 2D**). Also, an increase in Pseudomonadales to *Moraxella* was observed in OPV revaccinated (green nodes in **Figure 2C**), whereas an increase in Pasteurellales to *Haemophilus* was observed in the Control group (blue nodes in **Figure 2D**). Similar results were found after excluding antibiotic users in both groups (data not shown).

Supplementary Table 1. Table S1 describes house types regarding building material, sanitation structure (toilet, water source), drinking water (water source), cooking energy and kitchen place, hygiene measures (use of soap), contact with animals and household size, composition, and interactions. Significant differences between the Intervention group (with OPV revaccinated infants) and Control group (with not revaccinated infants) were observed regarding the average number of people less than 15 years old sharing the same house (4.9 *versus* 6.3, *p*=0.02, respectively), the water source and the cooking energy sources, pointing to higher use of tap water (10.9% versus 0%, p=0.03) and charcoal to cook (12.8% *versus* 0, *p*=0.03) in the Intervention group.

Supplementary Table 2. Table S2 describes clinical information on pregnancy and delivery mode from each group recorded at enrollment are described in. No significant differences were observed between groups regarding months of pregnancy (9 months on average in both groups, *p*=0.73), birth weight (3.1 Kg on average in both groups, *p*=0.83), birthplace (equally distributed between home and health center in both groups, *p*=0.25) and delivery mode (just vaginal in both groups). Also, no significant differences were seen concerning the relative frequencies of mothers and infants tested for HIV at pregnancy (42.6% in the Intervention group *versus* 43.2% in the Control group, *p*=0.95) and delivery (23.4% in the Intervention group *versus* 20.5% in the Control group, p=0.73), respectively, as well as mothers submitted to malaria prophylaxis (83.0% in the Intervention group versus 91.0% in the Control group, *p*=0.26) and affected by malaria disease at pregnancy (17.0% in the Intervention group *versu*s 14.0% in the Control group, *p*=0.69). Despite the high frequency of mothers covered by malaria prophylaxis, the average number of doses of intermittent preventive treatment in pregnancy (IPTp) administrated was low in both groups (2.0 in the Intervention group *versus* 1.9 in the Control group, *p*=0.40), as well as the average number of malaria episodes on the mothers that reported to have had malaria (1.9 in the Intervention group *versus* 1.5 in the Control group, *p*=0.35).

Supplementary Table 3. Table S3 describes feed history of participants at enrollment and follow-up and no significant differences were observed between groups. Breastfeeding coverage was very high

among all participants at both time points. The same was observed regarding porridge feeding and water administration, including median number of doses administrated per week. Regarding another sources of nutrients, fruits were introduced at follow-up, however, in a lower extent. No food allergy or atopy was reported.

Supplementary Table 4. Table S4 describes the history of hospitalizations, disease episodes and use of medicines by the participants, recorded at enrollment phase. According to enrollment interview, the frequency of previous hospitalizations was low in both groups (2.1%). No hospitalizations were observed in the month before the enrollment in both groups. Regarding the previous use of medicines, the most used was the non-steroidal anti-inflammatory Paracetamol (51.1% in the intervention group *versus* 46.8 in the Control group, p=0.52). Use of antibiotics, such as Amoxicillin, Cloxacillin (a combination of Clarithromycin and Amoxicillin) and Cotrimoxazole (a combination of Sulfamethoxazole and Trimethoprim) was low in both groups and no significant differences were observed in its use. Some infants in both groups have already experienced episodes of malaria infection (19.2% in the Intervention group *versus* 14.9% in the Control group; p=0.58) and diarrhea (40.4% in the Intervention group *versus* 34.0% in the Control group; p=0.52).

Supplementary Table 5. Table S5 describes changes in anthropometric parameters from enrollment to follow-up. Weight gain (512.3 g *versus* 401.1 g, p=0.19) and MUAC increase (4.4 mm *versus* 1.8 mm, p=0.17) did not differ significantly during the two months between the Intervention and Control groups, respectively. However, height increase was significantly lower in the Intervention than in the Control group (1.8 cm and 2.5 cm, respectively, p=0.04).

Supplementary Table 6. Table S6 describe participant profiles at enrollment and follow-up, after excluding infants in both groups that used antibiotics during follow-up.

Supplementary Table 7. Table S7 describes changes in anthropometric parameters from enrollment to follow-up, after excluding infants in both groups that used antibiotics during follow-up.

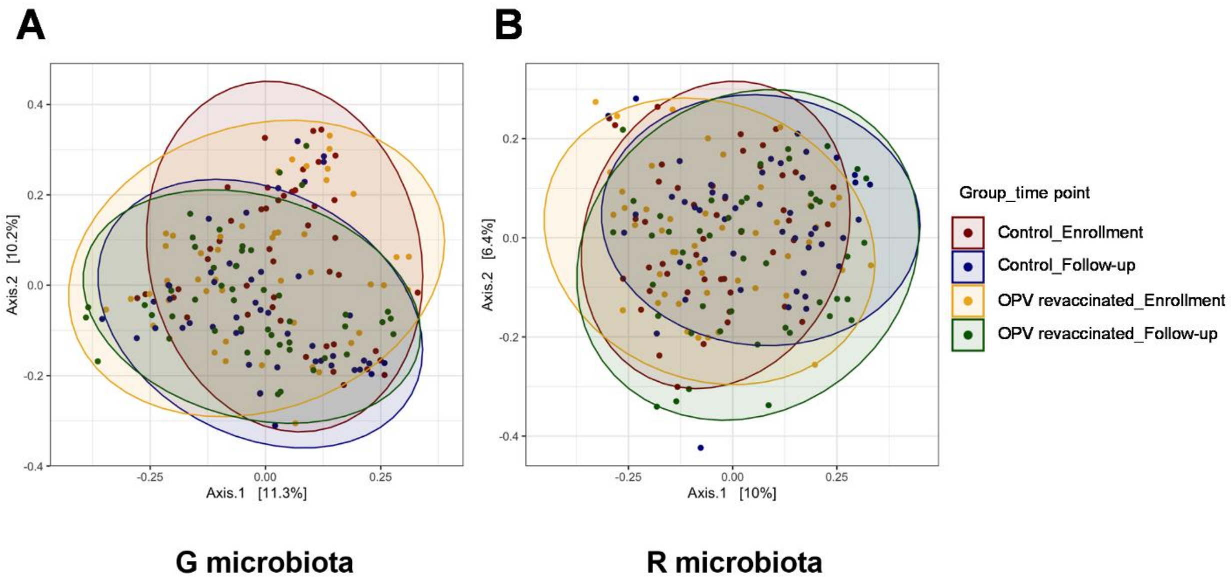

Supplementary Figure 1. PCoA based on Bray-Curtis dissimilarities for visualization of differences in bacterial composition between groups and time points for G and R microbiota samples. Samples for (**A**) G and (**B**) R microbiotas are displayed according to different group and time point combinations. PERMANOVA analyses showed that samples from both groups exhibited similar bacterial composition at enrollment and identified significant differences in bacterial composition over time for G and R microbiota samples in the Control group (**A**) and (**B**), bottom right corner, respectively) and for R microbiota samples in OPV revaccinated (**B**), top left corner. Heterogeneity in within-group variation over time in G microbiota samples in OPV revaccinated avoided a valid PERMANOVA analyses.

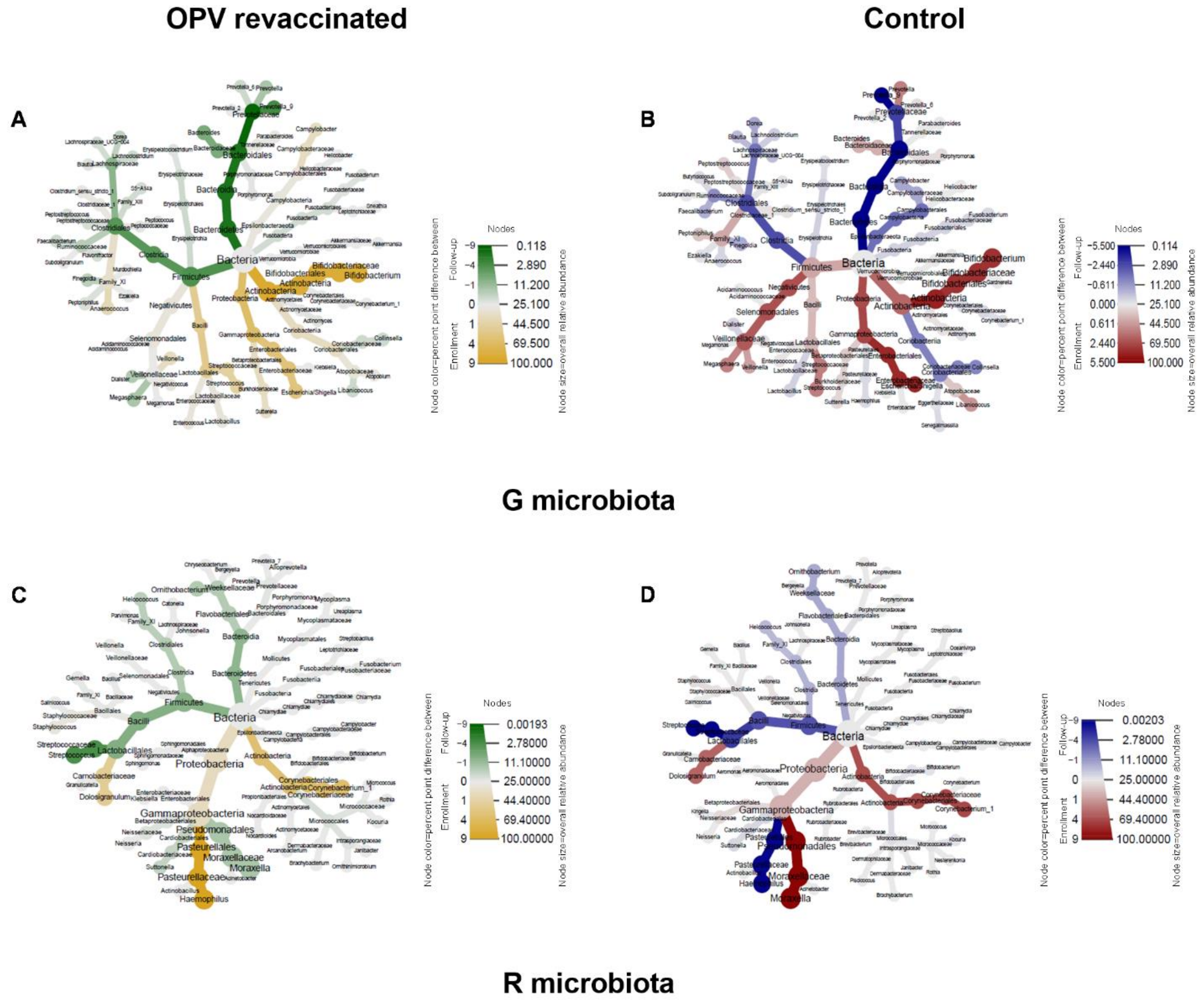

Supplementary Figure 2. Taxonomic heat trees based on relative abundance up to genus level within-groups over time for G and R microbiotas. Differences in relative abundance between enrollment and follow-up of the 50 most abundant genera and above within-group are shown. Nodes represent each taxon and the lines between them show the hierarchical relationships between taxa. Node colors indicate the percent point difference between enrollment and follow-up, allowing visualization of the most abundant taxa that decreased from enrollment to follow-up (yellow and red dark) and increased in the same period (green and blue) in OPV revaccinated and Control, respectively, for G (**A**) and R (**C**) microbiotas in OPV revaccinated, and (**B**) G and (**D**) R microbiotas in Control. Node sizes (diameters) indicate the overall relative abundance of each taxon at both time points.

Table S1. Description of household conditions of participants in each group

Characteristics	Intervention (n=47)	Control (n=47)	*P*
Parents literacy (reading)[a]			
Father	43 (91)	44 (94)	1.00
Mother	30 (64)	24 (51)	0.21
Type of house building material[a]			
Brickwork	42 (89)	42 (89)	1.00
Rammed earth	5 (11)	5 (11)	
Household[b]			
Number of residents per house			
Total	11/3-33/7	13/4-30/6	0.20
< 15 years old	5/1-15/3	6/1-15/3	**0.02**
Number of residents per room			
Total	3/2-7/1	3/2-6/1	0.67
< 15 years old	2/1-5/1	2/1-4/1	0.59
Animals[a]	42 (89)	46 (98)	0.20
Chicken	37 (79)	40 (85)	0.42
Pig	31 (66)	29 (62)	0.67
Goat	24 (51)	32 (68)	0.09
Dog	23 (49)	29 (62)	0.21
Others	21 (45)	18 (38)	0.53
*Toilet[a]			
Latrine	36 (77)	37 (80)	
Improved latrine	2 (4)	0	0.37
Without toilet	9 (19)	9 (20)	
Water source[a]			
Well	41 (89)	47 (100)	**0.03**
Tap water	5 (11)	0	
Use of soap[a]	9 (21)	4 (9)	0.10
Kitchen[a]			
Inside house	3 (6)	6 (13)	0.49
Outside house	44 (94)	41 (87)	
Cooking energy sources[a]			
Wood	41 (87)	47 (100)	**0.03**
Charcoal	6 (13)	0	

[a] n (%)
[b] average, minimum-maximum, standard deviation
* All without piped water. *P* P-value; differences between Intervention and Control groups were tested using Student's t-test or Mann-Whitney U test for quantitative data and Chi-square test or Exact Fisher test for qualitative data. Significant differences are displayed in bold.

Table S2. Clinical information of mothers' pregnancy and delivery of participants in each group

Characteristics	Intervention (n=47)	Control (n=47)	*P*
Months of pregnancy[a]	9.1/7-13/0.7	9.1/9-10/0.3	0.73
Birth weight (Kg)[a]	3.1/2.4-3.8/0.4	3-1/2-4-4.0/0.5	0.83
Birthplace[b]			
Home	19 (41)	25 (53)	0.25
Health centre	27 (59)	22 (47)	
Delivery mode[b]			
Vaginal	46 (100)	47 (100)	-
Caesarean	0	0	
Mother at pregnancy			
Tested for HIV[b]	20 (43)	19 (43)	0.95
Test results[b]			0.24
Positive	0	0	
Negative	18 (90.0)	14 (74)	
Undetermined	0	0	
Unknown	2 (10)	5 (26)	
Received prophylaxis to malaria[b]	39 (83)	40 (91)	0.26
Number of doses[a]	2/0-4/1	2/0-4/1	0.42
Had malaria[b]	8 (17)	6 (14)	0.69
Number of episodes[a]	2/1-3/1	1/1-2/1	0.35

Had other illnesses[b]	1 (2)	1 (2)	1.00
Infants at delivery			
Tested for HIV[b]	11 (23)	9 (20)	0.73
Test results[b]			1.00
Positive	0	0	
Negative	10 (91)	8 (89)	
Undetermined	0	0	
Unknown	1 (9)	1 (11)	

[a] average, minimum-maximum, standard deviation
[b] n (%)
P P-value; differences between Intervention and Control groups were tested using Student's t-test or Mann-Whitney U test for quantitative data and Chi-square test or Exact Fisher test for qualitative data. No significant differences were detected.

Table S3. Feeding history of participants at enrollment and follow-up interviews

Characteristics	Enrollment			Follow-up		
	Intervention (n=47)	Control (n=47)	*P*	Intervention (n=47)	Control (n=47)	*P*
*Breastfeeding[a]	47 (100)	47 (100)	1.00			
Age in month when stopping breastfeeding[b]	1.0/1.0-1.0	0	-			
Still breastfeeding[a]	46 (98)	47 (100)	1.00	46 (98)	47 (100)	1.00
Cow milk[a]	1 (2)	0	1.00	-	-	
Start at which month[b]	5/5-5	0	-			
Still drinking cow milk[a]	1 (2)	0	1.00			
Other foods[a]	37 (79)	35 (74)	0.63	47 (100)	45 (96)	0.50
Porridge[a]	28 (60)	28 (60)	1.00	41 (87)	40 (85)	0.77
Days a week[b]	7/7-7	7/7-7	1.00	7/7-7	7/7-7	1.00
Start at which month[b]	6/3-6	6/4-6	0.71	6./4-6	6/5-6	0.51
Fruits[a]	0	0	-	2 (4)	3 (7)	0.65
Days a week[b]	0	0	-	5/3-7	3/2-3	0.40
Start at which month[b]	0	0	-	6/6-6	6/6-6	0.80
Juices[a]	1 (2)	0	1.00	0	1 (2)	1.00
Days a week[b]	7/7-7	0	-	0	3/3-3	-
Start at which month[b]	6/6-6	0	-	0	7/7-7	-
Water[a]	34 (79)	30 (75)	0.66	47 (100)	45 (96)	0.50
Days a week[b]	7/7-7	7/7-7	1.00	7/7-7	7/7-7	1.00

Start at which month[b]	5/2-6	5/4-6	0.81	6/4-6	6/5-6	0.54
Food allergy[a]	0	0	-	0	0	-
Atopy[a]	0	0	-	0	0	-

* At follow-up interview, it was confirmed if the participant was breastfed at enrollment.
[a] n (%)
[b] median, interquartile range
P P-value; differences between Intervention and Control groups were tested using Student's t-test or Mann-Whitney U test for quantitative data and Chi-square test or Exact Fisher test for qualitative data. No significant differences were detected.

Table S4. History of hospitalizations, disease episodes and use of medicines recorded at enrollment interview

**Characteristics**[a]	**Intervention (n=47)**	**Control (n=47)**	***P***
Previous pneumonia infection	0	0	-
Previous tuberculosis infection	0	0	-
Previous malaria episode	9 (19)	7 (15)	0.58
Previous Diarrhoea	19 (40)	16 (34)	0.52
Hospitalization	1 (2)	1 (2)	1.00
Use of medicines	29 (63)	25 (54)	0.40
Vitamins	2 (4)	3 (6)	0.65
Paracetamol	24 (51)	22 (47)	0.52
Antibiotics	4 (8)	5 (11)	0.73
Amoxicillin	3 (75)	4 (80)	0.86
Cotrimoxazole	1 (25)	0	-
Cloxacillin	0	1 (20)	-
Antimalarial Coartem	1 (2)	0	-
Vaccines[b]	32 (68)	25 (53)	0.14
BCG	47 (100)	47 (100)	1.00
OPV (4)	32 (68)	25 (53)	0.14
Pentavalent (3)	37 (79)	31 (66)	0.17
Rotavirus (2)	43 (91)	38 (81)	0.14
•PCV13 (3)	36 (77)	31 (66)	0.25

••IPV (1)	3 (6)	3 (6)	1.00

[a] n (%)
[b] number of infants who completed the expected doses for the end of the 4th month of life
• Pneumococcal conjugate vaccine
••Inactivated poliomyelitis vaccine
P P-value; differences between Intervention and Control groups were tested using Chi-square test or Exact Fisher test for qualitative data. No significant differences were detected.

Table S5. Changes in anthropometric parameters over two months

**Characteristics**	**Intervention (n=47)**	**Control (n=47)**	***P***
Changes on anthropometric data[a]			
Weight gain (Kg)	0.5/-1,4-1,4/0.4	0.4/-1,2-1,6/0.5	0.27
Length increase* (cm)	2/-1-5/1	2/-0-6/2	**0.04**
MUAC increase (mm)	4/-12-54/10	2/-8-20/6	0.17
Time interval: enrollment – follow-up (months)	1.9/1.8-2.4/0.1	1.9/1.6-2.5/0.3	0.50
Weight gain/month (Kg)	0.3/-0.7-0.8/0.2	0.2/-0.7-0.8/0.2	0.30
Length increase/month* (cm)	1/-1-2/1	1/-0-3/1	**0.03**
MUAC increase/month (mm)	2/-6-2/5	1/-5-9/3	0.19

[a] average, minimum-maximum, standard deviation
*Two missing values in Intervention group and six missing values in Control group
P P-value; differences between Intervention and Control groups were tested using Student’s t-test or Mann-Whitney U test. Significant differences are displayed in bold.

Table S6. Participant profiles at enrollment and follow-up, after excluding infants in both groups that used antibiotics during follow-up

	Enrollment			Follow-up		
**Characteristics**	**Intervention (n=40)**	**Control (n=44)**	***P***	**Intervention (n=40)**	**Control (n=44)**	***P***
**Gender**[a]						
Male	16 (40)	22 (50)	0.48			
Female	24 (60)	22 (50)				
**Age (months)**[b]	6.83/6.06-7.80	6.35/5.11-7.31	0.12	8.78/7.90-9.73	8.26/7.03-9.20	0.11
**Anthropometric data**[c]						
Weight* (Kg)	7.5/ 5.3-9.9/1.0	7.1/4.5-10.1/1.0	0.11	7.9/5.4-11/1.2	7.5/5.0-11.2/1.3	**0.04**
Length** (cm)	68/61-74/3	66/61-72/3	**0.00**	69/60-75/3	68/61-74/3	**0.02**
MUAC (mm)	136/116-160/1	134/108-160/1	0.44	141/118-170/1	136/112-180/1	0.09
**Clinical signs at sample collection**						
Axillar temperature*** (°C)[c]	36.3/35.5-37.2/0.3	36.3/35.1-37.5/0.5	0.51	36.0/35.5-36.8/0.3	36.2/35.0-37.4/0.3	0.05
Urticaria[a]	0	0		0	0	
Cough[a]	9 (22)	15 (34)	0.35	8 (20)	15 (34)	0.23
Running nose – common cold[a]	24 (60)	24 (54)	0.78	19 (47)	21 (48)	1.00
Diarrhoea[a]	3 (7)	5 (11)	0.71	5 (12)	5 (11)	1.00

[a] number (%)

[b] median/Interquartile range
[c] average, minimum-maximum, standard deviation
*One missing value in Control group at enrollment (n=43) and six missing values in Intervention group at follow-up (n=34)
** One missing value in Intervention group (n=39) and six missing values in Control group (n=38) at enrollment
***One missing value in Intervention group (n=39) and one missing value in Control group (n=43) at enrollment and, six missing values in Intervention group (n=34) at follow-up
p P-value; differences between Intervention and Control groups were tested using Student's t-test or Mann-Whitney U test for quantitative data and Chi-square test or Exact Fisher test for qualitative data. Significant differences are displayed in bold.

Table S7. Changes in anthropometric parameters over two months, after excluding infants in both groups that used antibiotics in that period

**Characteristics**	**Intervention (n=40)**	**Control (n=44)**	***P***
Changes on anthropometric data[a]			
Weight gain (Kg)	0.5/-1.4-1.4/0.5	0.4/-1,2-1,6/0.5	0.24
Length increase (cm)	2/-1-5/1	2/-0.5-6/2	0.06
(MUAC) increase (mm)	5/-12-54/11	2/-8-20/6	0.10
Time interval: enrollment – follow-up (months)	1.9/1.7-2.4/0.1	1.9/1.5-2.4/0.2	0.50
Weight gain/month (Kg)	0.3/-0.7-0.8/0.2	0.2/-0.7-0.8/0.2	0.30
Length increase/month* (cm)	1/-1-2/1	1/-0-3/1	**0.03**
MUAC increase/month (mm)	2/-6-23/5	1/-5-9.0/3	0.11

[a] average, minimum-maximum, standard deviation
*One missing value in Intervention group (n=39) and six missing values in Control group (n=38)
p P-value; differences between Intervention and Control groups were tested using Student's t-test. Significant differences are displayed in bold.
